# Validity and reliability of a field technique for sweat Na^+^ and K^+^ analysis during exercise in a hot‐humid environment

**DOI:** 10.14814/phy2.12007

**Published:** 2014-05-11

**Authors:** Lindsay B. Baker, Corey T. Ungaro, Kelly A. Barnes, Ryan P. Nuccio, Adam J. Reimel, John R. Stofan

**Affiliations:** 1 Gatorade Sports Science Institute, Barrington, Illinois, USA

**Keywords:** Compact electrolyte analyzers, Horiba, regional sweat collection, sweat patches

## Abstract

This study compared a field versus reference laboratory technique for extracting (syringe vs. centrifuge) and analyzing sweat [Na^+^] and [K^+^] (compact Horiba B‐722 and B‐731, HORIBA vs. ion chromatography, HPLC) collected with regional absorbent patches during exercise in a hot‐humid environment. Sweat samples were collected from seven anatomical sites on 30 athletes during 1‐h cycling in a heat chamber (33°C, 67% rh). Ten minutes into exercise, skin was cleaned/dried and two sweat patches were applied per anatomical site. After removal, one patch per site was centrifuged and sweat was analyzed with HORIBA in the heat chamber (CENTRIFUGE HORIBA) versus HPLC (CENTRIFUGE HPLC). Sweat from the second patch per site was extracted using a 5‐mL syringe and analyzed with HORIBA in the heat chamber (SYRINGE HORIBA) versus HPLC (SYRINGE HPLC). CENTRIFUGE HORIBA, SYRINGE HPLC, and SYRINGE HORIBA were highly related to CENTRIFUGE HPLC ([Na^+^]: ICC = 0.96, 0.94, and 0.93, respectively; [K^+^]: ICC = 0.87, 0.92, and 0.84, respectively), while mean differences from CENTRIFUGE HPLC were small but usually significant ([Na^+^]: 4.7 ± 7.9 mEql/L, −2.5 ± 9.3 mEq/L, 4.0 ± 10.9 mEq/L (all *P* < 0.001), respectively; [K^+^]: 0.44 ± 0.52 mEq/L (*P* < 0.001), 0.01 ± 0.49 mEq/L (*P* = 0.77), 0.50 ± 0.48 mEq/L (*P* < 0.001), respectively). On the basis of typical error of the measurement results, sweat [Na^+^] and [K^+^] obtained with SYRINGE HORIBA falls within ±15.4 mEq/L and ±0.68 mEq/L, respectively, of CENTRIFUGE HPLC 95% of the time. The field (SYRINGE HORIBA) method of extracting and analyzing sweat from regional absorbent patches may be useful in obtaining sweat [Na^+^] when rapid estimates in a hot‐humid field setting are needed.

## Abstract (precis)

This study compared a field versus reference laboratory technique for extracting (SYRINGE vs. CENTRIFUGE) and analyzing sweat [Na^+^] and [K^+^] (compact HORIBA B‐722 and B‐731 versus ion chromatography, HPLC) collected with regional absorbent patches during exercise in a hot‐humid environment. The HORIBA analyzers provided highly reliable test‐retest and day‐to‐day measurements of sweat [Na^+^] and [K^+^]. The 95% limit of agreement between the SYRINGE HORIBA field technique and the reference laboratory‐based CENTRIFUGE HPLC technique was ±15.4 mEq/L and ±0.68 mEq/L for [Na^+^] and [K^+^], respectively; which may be acceptable in a field‐testing context, when simply aiming to estimate electrolyte losses for the purpose of identifying athletes/workers at greater risk for large electrolyte losses.

## Introduction

Replacing electrolytes, particularly sodium (Na^+^), lost from profuse sweating during/after physical activity in the heat may play an important role in preventing fluid/electrolyte imbalances (Shirreffs and Maughan 1998; Sawka et al. 2007; Shirreffs and Sawka 2011) and heat‐related whole‐body muscle cramps (Moss 1923; Talbott and Michelsen 1933; Bergeron 2003; Stofan et al. 2005; Armstrong et al. 2007) in sports and occupational settings. Sweat [Na^+^] can vary considerably among individuals [~20–80 mEq/L; (Allan and Wilson 1971; Shirreffs and Maughan 1997; Patterson et al. 2000)]. Thus, knowing an athlete/worker's sweat [Na^+^] could assist health professionals in planning personalized Na^+^ replacement strategies (Maughan and Shirreffs 2008; Armstrong and Casa 2009). The most accurate technique for determining sweat [Na^+^] is collection of sweat via whole‐body washdown (Shirreffs and Maughan 1997) followed by laboratory‐based electrolyte analysis (e.g., ion chromatography); however, these methods are impractical for field‐testing. A common field method for sweat collection is regional absorbent patches (Patterson et al. 2000; Baker et al. 2009); from which whole‐body washdown sweat [Na^+^] can be predicted using simple algorithms (Baker et al. 2009). However, there are currently limited practical options for [Na^+^] analysis of samples collected in the field.

The Horiba B‐722 is a compact, wireless, user‐friendly analyzer that uses ion‐selective electrode technology to derive measures of effective [Na^+^]. It is a commercially available device typically used for agriculture or food testing applications. One study tested the Horiba C‐122 (previous version of the B‐722) and found its measurement of sweat [Na^+^] to be reliable (CV = 3.7%) and accurate within 15 mEq/L (95% limits of agreement) of laboratory‐based ion chromatography (Goulet et al. 2012). The authors proposed that the Horiba [Na^+^] analyzer's reliability and accuracy was sufficient for use in field conditions where some degree of imprecision is acceptable (Goulet et al. 2012); for example, when the goal is simply to provide estimates of Na^+^ losses to guide Na^+^ replacement strategies. To further build on the development and validation of a field technique for sweat electrolyte analysis, it would be useful to determine the reliability and accuracy of the Horiba B‐722 [Na^+^] analyzer in hot‐humid conditions. It is important to determine the device's validity in these more extreme environmental conditions; as they are commonly encountered in field settings and represent a scenario where sweating rates and sweat electrolyte losses can be especially high.

Another challenge in conducting sweat electrolyte analysis in the field is extracting sweat sample from the regional absorbent patches. Typically, patches are centrifuged to extract the sweat sample for analysis (Baker et al. 2009; Dzidezic et al. 2014). A potential alternative technique that is more practical for field‐testing is using a syringe to compress the patch and collect the extracted sweat into a vial (Pahnke et al. 2010). However, syringe extraction of sweat has not been validated to date; sample volume may be limited using this technique and it remains unknown whether [Na^+^] is affected by the possibility of incomplete expulsion of sweat from the patch.

While Na^+^ is the primary electrolyte of interest, assessing sweat potassium (K^+^) losses can also be of value. For instance, to predict changes in serum [Na^+^] from mass balance equations measurements of sweat [K^+^] are needed as well as [Na^+^] (Kurtz and Nguyen 2003). In addition, measuring [K^+^] can be a useful quality control check of the sweat sample. Because there is little interindividual variation in sweat [K^+^], a sample with very high [K^+^] can be indicative of quality issues (e.g., sample evaporation, contamination, or leaching of electrolytes from skin (Weschler 2008x002F10)) that would not have been obvious from measuring sweat [Na^+^] alone. A Horiba B‐731 compact K^+^ analyzer is commercially available; however, its validity and reliability for measuring sweat samples has not been tested.

Using sweat collected with regional absorbent patches during exercise in a hot‐humid environment, the main objectives of this study were to (1) test the validity of the Horiba devices, by comparing the sweat [Na^+^] and [K^+^] obtained with Horiba B‐722 [Na^+^] and Horiba B‐731 [K^+^] analyzers (HORIBA) versus the reference laboratory‐based ion chromatography method (HPLC), (2) test the validity of the syringe sweat extraction method (SYRINGE), by comparing the sweat [Na^+^] and [K^+^] obtained with SYRINGE versus the reference laboratory‐based centrifuge method (CENTRIFUGE), and (3) test the validity of the combined field technique, by comparing the sweat [Na^+^] and [K^+^] obtained with SYRINGE HORIBA versus the reference laboratory technique for sweat extraction and analysis (CENTRIFUGE HPLC).

## Methods

### Subjects

Thirty healthy, moderately trained cyclists and triathletes (19 men, 11 women) took part in this study (age = 31 ± 6 years, height = 174 ± 8 cm, body mass = 69.3 ± 11.5 kg, VO_2max_ = 54.4 ± 6.8 mL·kg^−1^·min^−1^, HR_max_ = 186 ± 8 bpm). All participants gave their written informed consent before the study, which received approval from the Sterling Institutional Review Board (Atlanta, Georgia) for the protection of human subjects.

### Preliminary screening measurements

During their first visit to the laboratory**,** subjects’ nude body mass, height, resting heart rate, resting blood pressure, and 8‐h fasted blood glucose concentration were measured. Next, subjects completed a graded exercise test to assess cardiovascular fitness (12‐lead ECG, Schiller AT‐10 Plus, Schiller America; Doral, FL), maximal heart rate (HR_max_), and maximal aerobic capacity (VO_2max_) on a treadmill (Cosmed T200S, Cosmed USA; Chicago, IL) using the standard Bruce Protocol (ACSM 2008). VO_2max_ was measured using a MOXUS Metabolic system (AEI Technologies, Pittsburgh, PA).

### Experimental Procedures

At least 4 days after their preliminary screening visit, subjects reported to the laboratory for one experimental trial. Subjects reported to the lab at 08:30 h or 10:30 h and were asked to abstain from caffeine, alcohol, and vigorous exercise for 24 h, fast 2 h, and swallow an ingestible temperature sensor (HQ Inc; Palmetto, FL) 6–10 h before the trial. Subjects were also asked to drink ~500 mL of water 2 h before their trial to promote a well‐hydrated state at the start of exercise‐heat stress.

On arrival, subjects provided a urine sample to determine baseline pre‐exercise urine‐specific gravity (USG; digital refractometry, Model UG‐1, ATAGO; Bellevue, WA). A USG of < 1.020 g/mL (indicative of a well‐hydrated state) was required for continuation, otherwise trials were rescheduled. Next, subjects’ skin on the posterior forearms, forehead, upper back, upper chest, and anterior right thigh was cleaned with alcohol and shaved (if necessary) in preparation for later sweat patch application. Subsequently, subjects entered the environmental chamber (33.4 ± 0.6°C and 66.5 ± 2.1% relative humidity) and nude body mass was measured on a digital platform scale (Mettler Toledo; Columbus, OH) to the nearest gram. Baseline heart rate (Polar Electro; Lake Success, NY) and core temperature were recorded after 10 min of seated rest.

Subjects then mounted a stationary cycle ergometer (Velotron, Racermate Inc.; Seattle, WA), and cycled at 65–70% of HR_max_ (with fans placed in front of the subject for cooling, wind speed ~1.7 m/s). The duration of exercise was ~45–60 min depending upon subject's sweating rate (i.e., time needed for sweat collection). Subjects were allowed to drink water and/or sports drink ad libitum throughout the duration of exercise. Heart rate (Polar Electro; Lake Success, NY) and core temperature were monitored at 15‐min intervals throughout exercise. After exercise (i.e., after removal of all sweat patches) subjects dismounted the bike, voided their bladder, towel‐dried, and then a final body mass measurement was obtained. Total sweat loss was calculated from the change in pre to postexercise nude body mass, corrected for fluid intake and urine loss.

### Sweat collection

**Figure 1.**
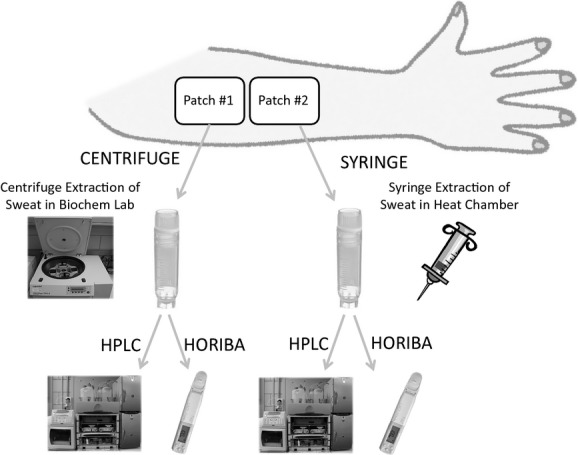
Sample extraction and analysis scheme.

After the onset of sweating (~10 min after the start of exercise), the subjects’ skin at each anatomical site was cleaned with deionized water and dried with gauze before patch application. Two sterile patches surrounded by an occlusive adhesive dressing (total of ~42 cm^2^ per patch; 3M Medical Sciences) were applied directly adjacent to each other at the following seven anatomical sites: (1) right anterior mid thigh, (2) right posterior mid forearm, (3) left posterior mid forearm, (4) upper chest, (5) right scapula, (6) left scapula, and (7) forehead. Patches were monitored throughout exercise and removed when sufficient sample was absorbed, but prior to complete saturation. Adjacent patches at each anatomical site were removed simultaneously. Upon removal with sterile tweezers, one of the patches at each site was designated for sweat extraction by CENTRIFUGE, and the other patch for sweat extraction via SYRINGE. The sample extracted by each method was subsequently analyzed for sweat [Na^+^] and [K^+^] by both HORIBA and HPLC. See Figure 1 for sample extraction and analysis scheme.

### Sweat extraction from patches

#### CENTRIFUGE (reference extraction method)

The CENTRIFUGE patches were transferred into an airtight plastic tube (Sarstedt Salivette separation filter) using sterile tweezers and centrifuged at 1811 *g* and 23°C for 10 min. Then, the sweat sample was aliquoted into cryovials and immediately analyzed with HPLC. The remaining sample was saved for subsequent analysis with HORIBA in the heat chamber.

#### SYRINGE (field extraction method)

After removal from the skin, the SYRINGE patches were immediately placed inside the barrel of a 5‐mL syringe using sterile tweezers. Then, an investigator depressed the syringe plunger to compress the patch, and collected the expelled sweat into a cryovial. Pilot work (with 18 sweat samples) showed that the volume of sweat sample extracted using this method is ~78% (63–87%) compared to traditional laboratory centrifugation of a sweat patch. The SYRINGE sweat samples were immediately analyzed in the heat chamber using HORIBA. The remaining sample was saved for subsequent analysis with HPLC.

### Sweat [Na^+^] and [K^+^] analysis

For each analysis method (HPLC and HORIBA) sweat samples were measured for both [Na^+^] and [K^+^] as long as sufficient sample volume was available. Sweat samples from a total of 210 possible sites were obtained. When there was limited sweat sample volume, measurement of [Na^+^] was prioritized over [K^+^]. Thus, the *n* for the method comparisons for sweat [Na^+^] is 210, whereas the *n* for sweat [K^+^] is 116. Sweat samples were measured in triplicate when sufficient sample volume was available or duplicate when volume was limited. The exact number of replicates for each method and each electrolyte are provided in the results section (Tables 1 and 2).

**Table 1.** Intraday reliability of HPLC and HORIBA analyzers for sweat [Na^+^] and [K^+^].

	Sweat [Na^+^]				Sweat [K^+^]			
	CENTRIFUGE HPLC		CENTRIFUGE HORIBA		CENTRIFUGE HPLC		CENTRIFUGE HORIBA	
	Replicate 1 vs. 2	Replicate 2 vs. 3	Replicate 1 vs. 2	Replicate 2 vs. 3	Replicate 1 vs. 2	Replicate 2 vs. 3	Replicate 1 vs. 2	Replicate 2 vs. 3
*n*	205	204	206	167	116	115	113	75
Mean Difference ± SD (mEq/L)	−0.20 ± 1.73	−0.09 ± 1.51	0.26 ± 1.41*	0.10 ± 1.18	0.01 ± 0.18	0.00 ± 0.15	−0.00 ± 0.14	−0.01 ± 0.15
95% CI of Mean Difference (mEq/L)	−0.44 to 0.04	−0.30 to 0.12	0.07 to 0.45	−0.08 to 0.28	−0.03 to 0.04	−0.03 ± 0.03	−0.03 to 0.02	−0.04 to 0.03
ICC	0.998*	0.999*	0.999*	0.999*	0.988*	0.992*	0.996*	0.996*
SEE (mEq/L)	1.73	1.51	1.38	1.18	0.18	0.15	0.14	0.15
TEM (mEq/L)	1.22	1.07	1.00	0.83	0.13	0.11	0.10	0.11
CV (%)	1.97	1.67	1.35	1.17	3.27	2.81	2.35	2.21

To determine intraday reliability samples were measured in triplicate when possible (depending on sample volume; exact *n* for each replicate comparison is provided). CV, coefficient of variation; HPLC, ion chromatography using the Dionex ICS‐3000; HORIBA, Horiba B‐722 for sweat [Na^+^] and Horiba B‐731 for sweat [K^+^]; ICC, intraclass correlation coefficient (based on two‐way mixed ANOVA, absolute agreement, average measures); SEE, standard error of the estimate; TEM, typical error of measurement; *P < 0.001.

**Table 2.** Interday reliability of HORIBA analyzer for sweat [Na^+^] and [K^+^].

	Sweat [Na^+^]		Sweat [K^+^]	
	Day 1 to day 2	Day 2 to day 3	Day 1 to day 2	Day 2 to day 3
Mean difference ± SD (mEq/L)	−0.07 ± 3.34	−0.08 ± 2.03	−0.04 ± 0.39	−0.09 ± 0.32
95% CI of mean difference (mEq/L)	−1.00 to 0.86	−0.65 to 0.48	−0.17 to 0.09	−0.18 to 0.01
ICC	0.995*	0.998*	0.945*	0.967*
SEE (mEq/L)	2.76	1.94	0.36	0.32
TEM (mEq/L)	2.36	1.43	0.28	0.23
CV (%)	3.72	2.26	7.37	5.46

Interday reliability was measured across three repeat days in 52 and 46 samples for sweat [Na^+^] and [K^+^], respectively. CV, coefficient of variation; HORIBA, Horiba B‐722 for sweat [Na^+^] and Horiba B‐731 for sweat [K^+^]; ICC, intraclass correlation coefficient (based on two‐way mixed ANOVA, absolute agreement, average measures); SEE, standard error of the estimate; TEM, typical error of measurement.

#### HPLC (reference analysis method)

All sample analysis with the HPLC took place in a biochemistry laboratory at room temperature. CENTRIFUGE sweat samples were immediately prepared and placed in the HPLC for analysis. After syringe extraction, the SYRINGE sweat samples sat at room temperature in the biochemistry laboratory for at least 30 min prior to analysis with HPLC. Sweat samples were then diluted 1:100 with 18 MΩ ultrapure water (Milli‐Q, Millipore, Billerica) to a total volume of 1 mL. The ion chromatography system consisted of the following Dionex equipment: ICS‐3000, Ion Pac CS12A column, CR‐TC, Ion Pac CG12A guard, CSRS 300 suppressor and conductivity detector (Thermo‐Fisher, Waltham). Methodology included a 25 *μ*L injection at a 1.0 mL/min flow rate for 15 min, 59 mA suppression, and 20 mmol/L methanesulfonic acid isocratic eluent. Four‐point calibration standards were prepared using Dionex combined 6‐cation standard stock solution. The calibration range of [Na^+^] and [K^+^] were 4–40 ppm and 0.5–5 ppm (i.e., 400–4000 ppm and 50–500 ppm undiluted), respectively. Validation samples were run daily and periodically throughout a given sequence.

#### HORIBA (field analysis method)

All sample analysis with HORIBA took place in the heat chamber (33°C, 67% rh). SYRINGE samples were analyzed immediately upon extraction. After centrifugation, the CENTRIFUGE sweat samples sat in the heat chamber for at least 30 min to allow equilibration with the simulated field conditions prior to analysis with HORIBA.

The Horiba B‐722 and Horiba B‐731 are handheld (55 g with battery; 164 mm × 29 mm × 20 mm), battery‐operated analyzers that use ion‐selective electrode technology to derive effective [Na^+^] and [K^+^], respectively. Each device contains a well with two sensors, the ion‐selective electrode and reference electrode, both of which need to be covered with sample for analysis. Placement of a Horiba sampling sheet in the well disperses the sample across the entire sensor pad, necessitating very small sweat sample volume. According to the manufacturer, the devices’ operating conditions are 5–40°C and ≤85% rh. The measurement range is 23–2300 ppm for the Horiba B‐722 Na^+^ analyzer and 39 to 3900 ppm for the Horiba B‐731 K^+^ analyzer, with an accuracy of ±10%. When the measured ion concentration is out of these ranges the device will still display a value (display range is 0–9900), but the value blinks as in indication that it should only be used as a guide. For both Horiba analyzers, the measurement resolution is 1 ppm for values 0–99 ppm, 10 ppm for 100–990 ppm, and 100 ppm for 1000–9990 ppm. For reporting results in this study, sweat [Na^+^] and [K^+^] were converted from ppm to mEq/L by dividing the ppm values by the molar mass of the ions (22.989 for Na^+^ and 39.098 for K^+^).

Before each trial the Horiba B‐722 and Horiba B‐731 were calibrated according to manufacturer instructions. A 2‐point calibration was performed with 150 ppm and 2000 ppm standard solutions for Horiba B‐722 Na^+^ analyzer. A 1‐point calibration was performed with 150 ppm standard solution for Horiba B‐731 K^+^ analyzer. Calibration was repeated frequently throughout testing (every ~10 measurements) to avoid instrument drift (Goulet et al. 2012). To analyze sweat samples, a sampling sheet was first placed in the well and then 40 *μ*L of sweat was pipetted onto the sampling sheet. After the well's light shield cover was placed in the closed position, the “measure” button was depressed and a reading of the ion concentration (in ppm) was displayed within 10 sec on a digital LCD screen. The sensor pads were rinsed with deionized water and dried gently with a tissue wipe between sweat samples.

For the interday reliability testing of the Horiba B‐722 and Horiba B‐731, sweat samples were refrigerated (8°C) overnight and then allowed to warm up to ambient temperature in the heat chamber prior to analysis.

### Statistical analysis

To determine the validity of the HORIBA analyzers, the syringe sweat extraction method, and the combined field technique (CENTRIFUGE HORIBA, SYRINGE HPLC, and SYRINGE HORIBA, respectively, vs. CENTRIFUGE HPLC) for measuring sweat [Na^+^] and [K^+^], coefficients of variation (CV), intraclass correlation coefficients (ICC; based on two‐way mixed ANOVA, absolute agreement, average measures), standard error of the estimate (SEE), and typical error of the measurement (TEM) were calculated. Linear regression analyses were used to generate equations to predict CENTRIFUGE HPLC sweat [Na^+^] and [K^+^] from the CENTRIFUGE HORIBA, SYRINGE HPLC, and SYRINGE HORIBA methods. Pearson product‐moment correlations were calculated to assess the relations between methods. ICCs, CVs, SEEs, and TEMs were used to determine intraday reliability of HORIBA and HPLC as well as interday reliability of HORIBA. All CVs were calculated using the TEM expressed as a percentage according to Hopkins. The significance level for all statistical tests was set at *α *= 0.05. All data are reported as means ± SD. Analyses were conducted using SPSS V. 21 (IBM; Armonk, NY).

## Results

Subjects pre‐exercise USG was 1.003 ± 0.003. During exercise, mean power output, heart rate, and core temperature were 120 ± 26 W, 137 ± 13 bpm, and 38.0 ± 0.4°C, respectively. The duration of exercise was 51 ± 7 min; during which time, total sweat loss, urine loss, and fluid intake were 959 ± 164 mL, 138 ± 114 mL, and 735 ± 362 mL, respectively. Sweating rate was 1145 ± 259 mL/h. Overall fluid balance, expressed as change in body mass from pre to postexercise was −0.34 ± 0.49%.

### Reliability

#### Intraday

The intraday reliability of the Horiba B‐722 Na^+^ analyzer, Horiba B‐731 K^+^ analyzer, and HPLC Dionex ICS‐3000 (using CENTRIFUGE extraction of patch sweat) is shown in Table 1. To determine intraday reliability samples were measured in triplicate when possible (depending upon sample volume). ICC, SEE, TEM, and CV data are provided for replicate 1 versus 2 and replicate 2 versus 3 (exact sample size for each replicate comparison is provided in Table 1). The ICCs of the HORIBA analyzers were highly significant (*P* < 0.001) and similar to that of the HPLC for both sweat [Na^+^] and sweat [K^+^] (≥ 0.99). The CV's also suggested good reliability for both HPLC and the HORIBA analyzers for sweat [Na^+^] (< 2%) and sweat [K^+^] (<3–4%). The SEE's and TEM's for sweat [Na^+^] were all <2 mEq/L for both HPLC and HORIBA. The SEE's and TEM's for sweat [K^+^] were all <0.2 mEq/L for both HPLC and HORIBA.

#### Interday

Interday reliability of the Horiba B‐722 Na^+^ and Horiba B‐731 K^+^ analyzers (using CENTRIFUGE extraction of patch sweat) was measured across three repeat days in 52 and 46 samples for sweat [Na^+^] and [K^+^], respectively; results are shown in Table 2. The ICCs were highly significant (*P* < 0.001) for both sweat [Na^+^] and [K^+^]. SEE's were <3 mEq/L for sweat [Na^+^] and ≤0.4 mEq/L for sweat [K^+^]. Similarly, the TEM's were <3 mEq/L for sweat [Na^+^] and ≤0.3 for sweat [K^+^]. The CV's were somewhat lower for sweat [Na^+^] (~2–4%) than sweat [K^+^] (~5–7%).

### Validity

#### Validity of Horiba analyzers: CENTRIFUGE HORIBA versus CENTRIFUGE HPLC

**Figure 2.**
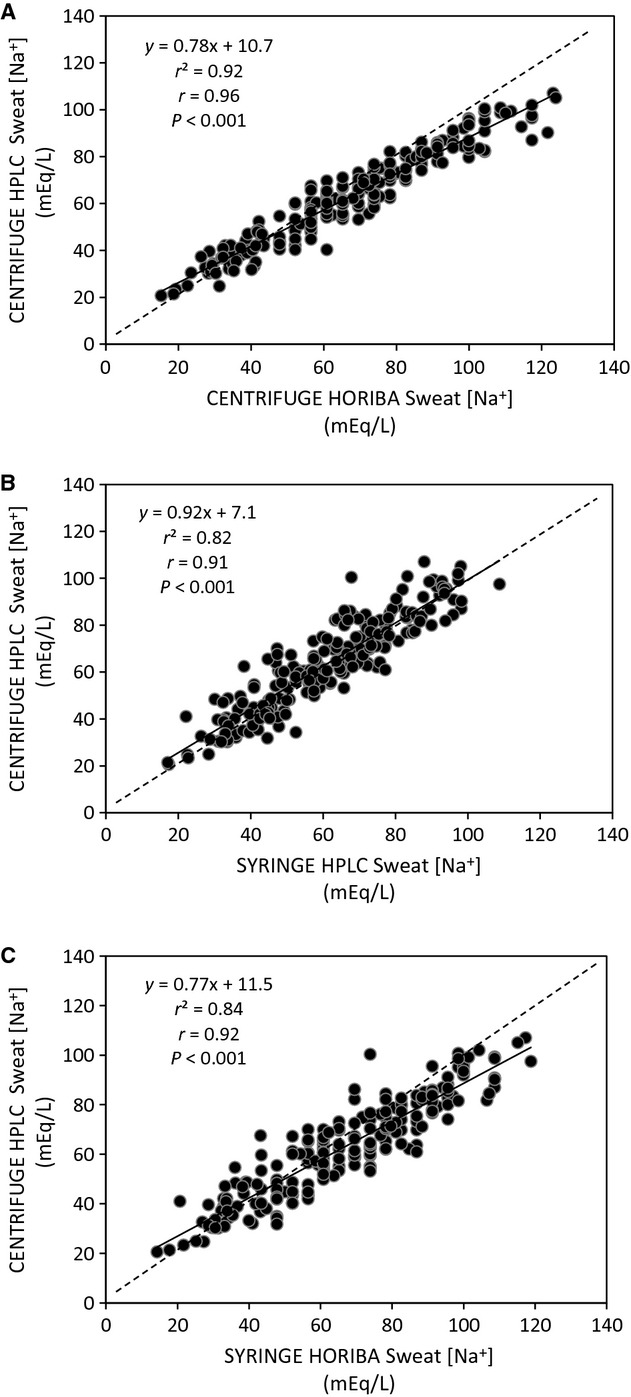
Regression of CENTRIFUGE HORIBA (centrifuge sweat extraction, compact Horiba B‐722 analysis), SYRINGE HPLC (syringe sweat extraction, ion chromotography analysis), and SYRINGE HORIBA (combined field technique using syringe sweat extraction and compact Horiba B‐722 analysis) on CENTRIFUGE HPLC (reference laboratory technique using centrifuge sweat extraction and ion chromatography analysis) for sweat [Na^+^]. *n* = 210. Dashed line is the line of identity.

**Figure 3.**
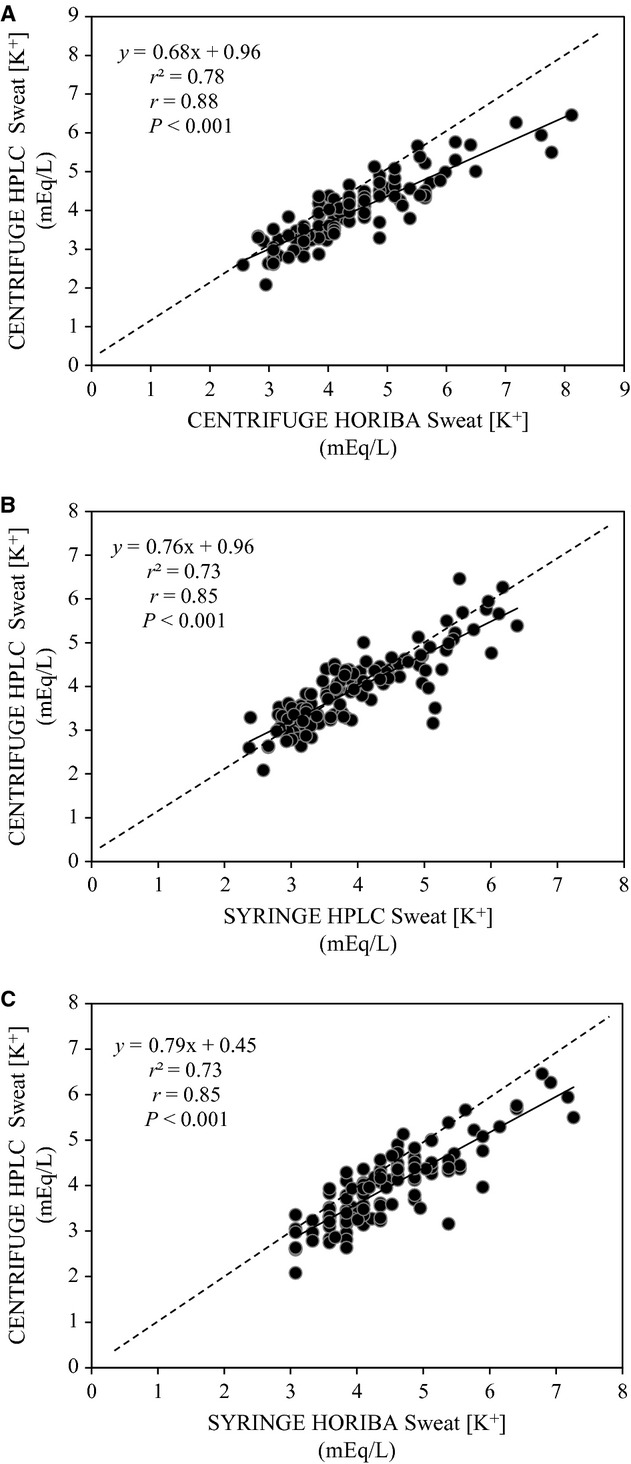
Regression of CENTRIFUGE HORIBA (centrifuge sweat extraction, compact Horiba B‐731 analysis), SYRINGE HPLC (syringe sweat extraction, HPLC analysis), and SYRINGE HORIBA (combined field technique using syringe sweat extraction and compact Horiba B‐731 analysis) on CENTRIFUGE HPLC (reference laboratory technique using centrifuge sweat extraction and ion chromatography analysis) for sweat [K^+^]. *n* = 116. Dashed line is the line of identity.

Table 3 shows validity results for the Horiba B‐722 Na^+^ analyzer and Horiba B‐731 K^+^ analyzer. The ICCs between CENTRIFUGE HORIBA and CENTRIFUGE HPLC were highly significant (*P* < 0.001) for both sweat [Na^+^] (0.96) and [K^+^] (0.87). However, mean sweat [Na^+^] and [K^+^] obtained with CENTRIFUGE HORIBA significantly overestimated that of CENTRIFUGE HPLC by 4.6 ± 7.8 and 0.4 ± 0.5 mEq/L, respectively. The TEM results suggested that sweat [Na^+^] obtained with CENTRIFUGE HORIBA falls within ±5.5 mEq/L of the CENTRIFUGE HPLC value 68% of the time and within ±11.0 mEq/L of the CENTRIFUGE HPLC value 95% of the time. For sweat [K^+^] the TEM results suggested that the CENTRIFUGE HORIBA value falls within ±0.4 mEq/L of the CENTRIFUGE HPLC value 68% of the time and within ±0.8 mEq/L of the CENTRIFUGE HPLC value 95% of the time. The regression of CENTRIFUGE HORIBA on CENTRIFUGE HPLC is provided in Figures 2A and 3A for sweat [Na^+^] and [K^+^], respectively. The relation between CENTRIFUGE HORIBA and CENTRIFUGE HPLC was highly significant (*P* < 0.001) for both sweat [Na^+^] (*r* = 0.96) and [K^+^] (*r* = 0.88).

**Table 3.** Validity of Horiba analyzer: CENTRIFUGE HORIBA versus CENTRIFUGE HPLC for sweat [Na^+^] and [K^+^].

	Sweat [Na^+^]	Sweat [K^+^]
Mean difference ± SD (mEq/L)	4.71 ± 7.87*	0.44 ± 0.52*
95% CI of mean difference (mEq/L)	3.64–5.78	0.34–0.54
ICC	0.96*	0.87*
SEE (mEq/L)	5.56	0.39
TEM (mEq/L)	5.56	0.37
CV (%)	9.37	8.36

*n* = 210 for sweat [Na^+^] and 116 for sweat [K^+^]. CV. coefficient of variation; CI, confidence interval; HPLC, ion chromatography using the Dionex ICS‐3000; HORIBA, Horiba B‐722 for sweat [Na^+^] and Horiba B‐731 for sweat [K^+^]; ICC, intraclass correlation coefficient (based on two‐way mixed ANOVA, absolute agreement, average measures); SD, standard deviation; SEE, standard error of the estimate; TEM, typical error of measurement; *P < 0.001.

#### Validity of syringe sweat extraction: SYRINGE HPLC versus CENTRIFUGE HPLC

Table 4 shows validity results for the syringe sweat extraction method. The ICCs between SYRINGE HPLC and CENTRIFUGE HPLC were highly significant (*P* < 0.001) for both sweat [Na^+^] (0.94) and [K^+^] (0.92). However, mean sweat [Na^+^] obtained with SYRINGE HPLC significantly underestimated that of CENTRIFUGE HPLC by 2.5 ± 9.3 mEq/L. The TEM results suggested that sweat [Na^+^] obtained with SYRINGE HPLC falls within ±5.5 mEq/L of the CENTRIFUGE HPLC value 68% of the time and within ±11.0 mEq/L of the CENTRIFUGE HPLC value 95% of the time. For sweat [K^+^] the TEM results suggested that the SYRINGE HPLC value falls within ±0.4 mEq/L of the CENTRIFUGE HPLC value 68% of the time and within ±0.8 mEq/L of the CENTRIFUGE HPLC value 95% of the time. The regression of SYRINGE HPLC on CENTRIFUGE HPLC is provided in Figures 2B and 3B for sweat [Na^+^] and [K^+^], respectively. The relation between SYRINGE HPLC and CENTRIFUGE HPLC was highly significant (*P* < 0.001) for both sweat [Na^+^] (*r* = 0.91) and [K^+^] (*r* = 0.85).

**Table 4.** Validity of syringe sweat extraction: SYRINGE HPLC versus CENTRIFUGE HPLC for sweat [Na^+^] and [K^+^].

	Sweat [Na^+^]	Sweat [K^+^]
Mean difference ± SD (mEq/L)	−2.52 ± 9.25*	0.01 ± 0.49
95% CI of mean difference (mEq/L)	−3.78 to −1.26	−0.08 to 0.10
ICC	0.94*	0.92*
SEE (mEq/L)	9.07	0.50
TEM (mEq/L)	5.51	0.35
CV (%)	11.20	9.22

*n* = 210 for sweat [Na^+^] and 116 for sweat [K^+^]. CV. coefficient of variation; CI, confidence interval; HPLC, ion chromatography using the Dionex ICS‐3000; HORIBA, Horiba B‐722 for sweat [Na^+^] and Horiba B‐731 for sweat [K^+^]; ICC, intraclass correlation coefficient (based on two‐way mixed ANOVA, absolute agreement, average measures); SD, standard deviation; SEE, standard error of the estimate; TEM, typical error of measurement; *P < 0.001.

#### Validity of combined field technique: SYRINGE HORIBA versus CENTRIFUGE HPLC

Table 5 shows validity results for the combined field technique; that is, syringe sweat extraction method followed by Horiba analysis. The ICCs between SYRINGE HORIBA and CENTRIFUGE HPLC were highly significant (*P* < 0.001) for both sweat [Na^+^] (0.93) and [K^+^] (0.84). However, mean sweat [Na^+^] and [K^+^] obtained with SYRINGE HORIBA significantly overestimated that of CENTRIFUGE HPLC by 4.0 ± 10.9 mEq/L and 0.5 ± 0.5 mEq/L, respectively. The TEM results suggested that sweat [Na^+^] obtained with SYRINGE HORIBA falls within ±7.7 mEq/L of the CENTRIFUGE HPLC value 68% of the time and within ±15.4 mEq/L of the CENTRIFUGE HPLC value 95% of the time. For sweat [K^+^] the TEM results suggested that the SYRINGE HORIBA value falls within ±0.3 mEq/L of the CENTRIFUGE HPLC value 68% of the time and within ±0.6 mEq/L of the CENTRIFUGE HPLC value 95% of the time. The regression of SYRINGE HORIBA on CENTRIFUGE HPLC is provided in Figures 2C and 3C for sweat [Na^+^] and [K^+^], respectively. The relation between SYRINGE HORIBA and CENTRIFUGE HPLC was highly significant (*P* < 0.001) for both sweat [Na^+^] (*r* = 0.92) and [K^+^] (*r* = 0.85).

**Table 5.** Validity of field technique using syringe extraction of sweat and compact Horiba analysis: SYRINGE HORIBA versus CENTRIFUGE HPLC for sweat [Na^+^] and [K^+^].

	Sweat [Na^+^]	Sweat [K^+^]
Mean difference ± SD (mEq/L)	3.97 ± 10.87*	0.50 ± 0.48*
95% CI of mean difference (mEq/L)	2.49–5.45	0.42–0.59
ICC	0.93*	0.84*
SEE (mEq/L)	8.68	0.44
TEM (mEq/L)	7.68	0.34
CV (%)	12.54	8.48

*n* = 210 for sweat [Na^+^] and 116 for sweat [K^+^]. CV. coefficient of variation; CI, confidence interval; HPLC, ion chromatography using the Dionex ICS‐3000; HORIBA, Horiba B‐722 for sweat [Na^+^] and Horiba B‐731 for sweat [K^+^]; ICC, intraclass correlation coefficient (based on two‐way mixed ANOVA, absolute agreement, average measures); SD, standard deviation; SEE, standard error of the estimate; TEM, typical error of measurement; *P < 0.001.

## Discussion

The main findings from this study were as follows: (1) there was high intraday and interday reliability of the Horiba B‐722 Na^+^ and Horiba B‐731 K^+^ analyzers, (2) sweat [Na^+^] and [K^+^] obtained with the SYRINGE HORIBA field technique was significantly correlated with that of the reference laboratory‐based CENTRIFUGE HPLC method, (3) on average, SYRINGE HORIBA overestimated CENTRIFUGE HPLC sweat [Na^+^] by 4.0 mEq/L and sweat [K^+^] by 0.50 mEq/L, and (4) based on typical error of the measurement results, sweat [Na^+^] and [K^+^] obtained with SYRINGE HORIBA falls within ±7.7 mEq/L and ±0.34 mEq/L, respectively, of CENTRIFUGE HPLC 68% of the time and within ±15.4 mEq/L and ±0.68 mEq/L, respectively, of CENTRIFUGE HPLC 95% of the time.

Na^+^ replacement stimulates thirst, promotes whole‐body and extracellular fluid retention, and helps maintain/restore electrolyte balance (Nose et al. 1988; Shirreffs and Maughan 1998; Sawka et al. 2007). Na^+^ intake during exercise is recommended when fluid and Na^+^ losses from sweating are high (e.g., >3–4 g of Na^+^) or when exercise lasts more than ~2 h (Coyle 2004; Shirreffs and Sawka 2011). After exercise, the goal is to completely replace any fluid and Na^+^ deficit (Shirreffs and Maughan 1998; Shirreffs et al. 2004; Shirreffs and Sawka 2011). It is well known that there are large interindividual differences in sweat [Na^+^] (Allan and Wilson 1971; Shirreffs and Maughan 1997; Patterson et al. 2000). Thus, having a tool to measure an athlete/worker's sweat [Na^+^] could assist health professionals in planning more personalized Na^+^ replacement strategies (Maughan and Shirreffs 2008; Armstrong and Casa 2009). In most situations, sports teams and industries do not have the resources for testing in a controlled laboratory setting. Thus, practitioners need an inexpensive, user‐friendly method to estimate sweat [Na^+^] losses on the field of sports play or in an industrial setting. The results of this study suggest that the SYRINGE HORIBA method of extracting and analyzing sweat from regional absorbent patches may be useful in obtaining sweat [Na^+^] when rapid estimates in a hot‐humid field setting are needed.

The regional absorbent patch method is commonly used to collect sweat in the field (Maughan et al. 2004; Stofan et al. 2005; Kilding et al. 2009; Pahnke et al. 2010); however, to date there is limited data on field techniques for analyzing regional sweat [Na^+^] and [K^+^] (Boisvert and Candas 1994; Goulet et al. 2012). In this study, the Horiba B‐722 Na^+^ and Horiba B‐731 K^+^ analyzers were highly reliable during replicate measures of samples within‐day and from day‐to‐day. The 95% limits of agreement relating SYRINGE HORIBA to the reference laboratory‐based CENTRIFUGE HPLC technique was 15.4 mEq/L and 0.68 mEq/L for sweat [Na^+^] and [K^+^], respectively. Thus, the SYRINGE HORIBA technique may be used in a relative sense to track changes in sweat [Na^+^] within individuals in different environmental conditions or with changes in training or heat acclimatization status. This technique (along with information on sweating rate) may also be useful in identifying which individuals on a sports team or work group are at risk for higher sweat Na^+^ losses. For example, one study reported that football players with a history of heat cramps had a sweat [Na^+^] and total sweat Na^+^ loss more than twice that of players with no history of heat cramps (55 ± 16 mEq/L vs. 25 ± 10 mEq/L) (Stofan et al. 2005). With a 95% limit of agreement of 15.4 mEq/L, the SYRINGE HORIBA method should be sensitive enough to differentiate between the two groups in this example. The SYRINGE HORIBA technique also provides the advantage of allowing immediate feedback to the athlete/worker as opposed to having to wait until sweat samples are sent back to the laboratory for electrolyte analysis.

In this study we also compared SYRINGE HPLC versus CENTRIFUGE HPLC and CENTRIFUGE HORIBA versus CENTRIFUGE HPLC to test the validity of the syringe sweat extraction technique and the Horiba electrolyte analysis separately. These comparisons produced similar ICCs, CVs and TEMs. However, the paired differences between SYRINGE HPLC versus CENTRIFUGE HPLC (−2.5 mEq/L for [Na^+^] and +0.01 mEq/L for [K^+^]) and CENTRIFUGE HORIBA versus CENTRIFUGE HPLC (+4.7 mEq/L for [Na^+^] and +0.44 mEq/L for [K^+^]) suggest that the Horiba analysis accounts for most of the offset between SYRINGE HPLC and CENTRIFUGE HPLC.

One previous study has tested the validity and reliability of a Horiba Na^+^ analyzer; with results similar to that of the present investigation. Using sweat samples obtained with the regional absorbent patch method, Goulet et al. (2012) found that the Horiba C‐122 produced reliable measures of sweat [Na^+^] (intraday CV of 3.7%, interday CV of 0.7%). Compared to ion chromatography analysis, the Horiba C‐122 had a mean bias of +1.7 mEq/L and the 68% limit of agreement was ±7.5 mEq/L. The discrepancy in sweat [Na^+^] measured with Horiba analyzers versus the reference ion chromatography method could be due to a number of factors. As mentioned previously, the Horiba analyzers are primarily intended for agricultural (soil) and food testing applications. Thus, the control solutions (150 and 2000 ppm for both Na^+^ and K^+^) and corresponding 2‐point calibration curve are not specific to sweat analysis. This may explain why the offset between HORIBA and HPLC in this study was especially apparent at very high [Na^+^] (e.g., above ~2300 ppm or ~100 mEq/L) (see Fig. 2A). Other potential explanations may be related to interference of the Na^+^ or K^+^ electrodes by other ionic species present in sweat (e.g., ammonia, lactate, calcium, magnesium, etc.) or the lower measurement resolution of the Horiba analyzers than the HPLC. Others have also reported variability in results obtained when using direct (e.g., ion chromatography, flame photometry) versus indirect (ion‐selective electrode) measures of [Na^+^] (Dzidezic et al. 2014). The statistically significant overestimation of sweat [Na^+^] and[K^+^] using SYRINGE HORIBA makes this technique inadequate when precise measurements of sweat electrolyte concentration are desired (e.g., mass balance studies). In these cases, a laboratory‐based method is more appropriate.

Because of the significant correlation between SYRINGE HORIBA and CENTRIFUGE HPLC, regression equations can be used to predict regional sweat [Na^+^] and [K^+^] from SYRINGE HORIBA. It is well accepted that regional methods overestimate sweat [Na^+^] compared with that obtained with the criterion whole‐body washdown method (Shirreffs and Maughan 1997; Patterson et al. 2000; Baker et al. 2009). Thus, to obtain the most accurate sweat [Na^+^] measurements the whole‐body washdown method should be employed. If the whole‐body washdown method is not a viable option, both factors (offset between regional vs. whole‐body washdown (Baker et al. 2009) and SYRINGE HORIBA vs. CENTRIFUGE HPLC) should be incorporated into the algorithms to predict whole‐body [Na^+^] from SYRINGE HORIBA.

### Future directions

There are several gaps in the literature related to electrolyte loss and replacement in athletes. For instance, there are currently no normative data available to categorize athletes as having low, moderate, and high sweat [Na^+^]. To date, the only reference ranges available for sweat [Na^+^] are for clinical use in the diagnosis of cystic fibrosis (Hammond et al. 1994). While Na^+^ intake is recommended during prolonged exercise and/or heavy sweating (Coyle 2004; Shirreffs and Sawka 2011), it is not clear exactly how much Na^+^ replacement (e.g., 50% or 100%) is necessary for maintenance of performance or avoidance of heat‐related issues. Having this information will help determine whether the overestimation of sweat [Na^+^] using the SYRINGE HORIBA field technique has a practically significant effect on categorizing athletes sweat [Na^+^] or electrolyte replacement recommendations.

## Summary

This study compared a field versus reference laboratory technique for extracting (SYRINGE vs. CENTRIFUGE) and analyzing [Na^+^] and [K^+^] (compact Horiba B‐722 and B‐731, HORIBA vs. ion chromatography, HPLC) from sweat collected with regional absorbent patches during exercise in a hot‐humid environment. The Horiba B‐722 and Horiba B‐731 analyzers provided highly reliable measurements of sweat [Na^+^] and [K^+^], respectively. The 95% limit of agreement between the SYRINGE HORIBA field technique and the reference laboratory‐based CENTRIFUGE HPLC technique was ±15.4 mEq/L and ±0.68 mEq/L for [Na^+^] and [K^+^], respectively; which may be acceptable when simply aiming to estimate electrolyte losses for the purpose of identifying athletes/workers at greater risk for large electrolyte losses. Taken together, these study results indicate that the SYRINGE HORIBA method of extracting and analyzing sweat from absorbent patches may assist clinicians in making rapid estimations of regional sweat [Na^+^] and [K^+^] of athletes/workers in a hot‐humid field setting.

## Acknowledgments

The authors thank the study participants for their time and effort. They also thank Horiba, Ltd for technical assistance and Dennis Passe, Ph.D. for statistical consultation. Financial support for this study was provided by the Gatorade Sports Science Institute, a division of PepsiCo, Inc.

## Conflict of Interest

Authors Lindsay B. Baker, Corey T. Ungaro, Kelly A. Barnes, Ryan P. Nuccio, Adam J. Reimel, and John R. Stofan are employees of the Gatorade Sports Science Institute, a division of PepsiCo, Inc. The views expressed in this article are those of the authors and do not necessarily reflect the position or policy of PepsiCo, Inc.
